# The American Medical Association Plan of Reorganization*Read before the first regular meeting of the Bexar County Medical Society, August 6, 1903.

**Published:** 1903-09

**Authors:** James Hall Bell

**Affiliations:** San Antonio, Texas


For Texas Medical Journal.

### The American Medical Association Plan of Reorganization.*

BY JAMES HALL BELL, M. D., SAN ANTONIO, TEXAS.

I have been asked to open for discussion the question of the reorganization of the medical profession of the United States. As a

*Read before the first regular meeting of the Bexar County Medical Society, August 6, 1903.

preliminary note, may I, in a few words, characterize its disorganization? For more than a hundred years the profession of medicine has been conspicuously without influence in the enactment and control of legislation vitally touching its general welfare, and utterly unable or unwilling to put in operation the machinery necessary to a wise and economic administration of its own internal affairs. The general disorder and confusion everywhere visible in the profession, its internal feuds and hatreds and consequent formation of cliques in every community, the widespread prevalence of personal spites and scandals, which created suspicion between honest and capable men and drove them into professional solitude, have marred the really great achievements of medicine, crippled its followers in every material interest, and brought them into open disrepute before the world.

We have not been deliberately wicked in these respects, but criminally negligent; and I feel satisfied, in my own mind, that any one who will give the subject thoughtful and dispassionate consideration will inevitably be led to the illuminating conclusion that many, if not all, of our disorders are the results of disunion. Our profession, the greatest of all professions in its aspirations and accomplishments, has never been properly organized. To this single fact, I repeat, is justly attributable all our woes; and everyone knows, from personal observation and experience not always free from bitterness, that we have had woes in abundance.

For some years the question of the reorganization of the medical profession of the United States has been uppermost in the minds of many of the leading men of the American Medical Association. It is easier, however, to discover the existence of a defect than it is to find and apply the right remedy. It was at once apparent, for instance, that difficulties of a negative character had to be overcome; ignorance, prejudice and old fogyism faced and fought, and the old structure torn down before the new edifice could be reared in its place. It was realized, moreover, that the new building, in the face of hostile and persistent criticism, would have to be constructed on a large and lasting plan, a plan large enough to accommodate, within defined limits, every legalized practitioner of medicine in the United States, strong enough to induce all eligible men to accept it as a place of permanent residence, and so justly apportioned that every dweller therein should have his equal rights forever guaranteed. With these preliminaries generally understood, if not agreed upon, the work of actual reconstruction began.

Three years ago, at the Atlantic City meeting, the American Medical Association appointed a Committee on Reorganization. This committee drafted and presented at the Saratoga meeting, a year later, a new constitution and by-laws for the American Medical Association itself. They were adopted. The members of this committee were reappointed, this time a Committee on Organization, and authorized to frame a constitution and by-laws for State and county societies. Thirty States, approximately, our own included, have already formally adopted the constitution and by-laws proposed by this committee; they are now actively employed in organizing their component county societies. Other States are falling in line as fast as practicable. The reorganization of the medical profession of the United States may, therefore, be regarded as an accomplished fact. The benefits to the profession, and to society at large, of the new organization are already apparent. In time they will be immense.

This new organization is nearly perfect in its provisions and operations. It brings the entire regular profession of the country into compact and harmonious relations and assures the permanency of these relations. It separates the business and scientific departments in organization work, and provides for national, State and county societies a system of just representation, as free from politics as human foresight and ingenuity could devise. In practice it makes no difference whether the question at issue affects a person or a principle, whether it is special to the individual or general to the profession, the national, State and county organizations stand together as a unit, ready, willing and able to redress what is wrong and to defend what is right. I am, therefore, not dreaming when I affirm that in a very few years our profession will have ample power (it already has more than the disposition) to compel legislation in the right direction. This means, among other things, that we will have laws, uniform in all the counties and States, governing the practice of medicine, medical education, quarantine, sanitation and hygiene; that a properly qualified man will be permitted to practice his profession anywhere in this country without reference to State lines—that is to say, inter-state reciprocity. It may ultimately mean international reciprocity, for I notice, in this week's issue of the *Journal*, that the Swedish Medical Association has recently been reorganized on the American plan. It means certainly the abolition of the quack. That ugly and mischievous excrescence is already shivering and shrinking in the malodorous gloom of approaching dissolution. Even the medical politician, the chronic applicant for office, the patient and adroit seeker after votes, is not quite easy in his mind. He, too, has trouble of his own to occupy him largely, if not exclusively. He must qualify for larger horizons than the gratification of exclusively selfish ambitions.

Thus far I have sketched as rapidly as I could the larger and outer aspects of the American Medical Association's plan of organization, and without anticipating what Dr. Russ and others may have to say relative to its minute mechanism, will note without comment a few cardinal points for further discussion:

1. The national, State and county organizations are intercurrent in their operations, and inseparably bound together. Each of these societies has its appointed work to do, but the work itself is complementary—a necessary part of the common possession, shared by all.

2. Unquestionably the most important part of this work is performed by county societies. They not only furnish all the members of national and State associations, but pass on the individual social and professional qualifications of each member.

3. No man not a member of the medical society of the county in which he lives is eligible for membership in either State or national organization.

4. The delegates of county societies unite to form the House of Delegates of the State Medical Association, and the House of Delegates of State associations elect delegates to form the House of Delegates of the American Medical Association.

5. The right of appeal to the House of Delegates of the national association is assured to each member, but the rules of procedure are under constitutional prescription. The case must reach this court of last appeal through the county and State societies, in the order named.

6. Each member has guaranteed him equal rights and privileges under the laws of each of the three organizations, and these laws are based on the Golden Rule. They are in no instance or manner prescriptive of what is right in professional conduct, but they oppose with strong and forbidding hands wrong principles and actions of every character.

In a word, and finally, the supreme desire of reorganization work is not to weaken professional character, but to strengthen and build it up; not to curtail professional liberty, but to give it large, uniform scope in a better atmosphere; not to isolate men as enemies, but to draw them together as friends, and thus subserve their own as well as the higher interests of science.

The union of the profession has been accomplished, and the necessary machinery put in operation to secure these conditions and to perpetuate them as the essential underlying principles of the professional character in this country.

